# Acute Shortening and Re-Lengthening (ASRL) in Infected Non-union of Tibia - Advantages Revisited

**DOI:** 10.5704/MOJ.2007.012

**Published:** 2020-07

**Authors:** RK Baruah, JP Baruah, S Shyam-Sunder

**Affiliations:** Department of Orthopaedics, Assam Medical College Hospital, Dibrugarh, India

**Keywords:** Ilizarov, gap non-union, internal bone transport, ASRL

## Abstract

**Introduction:**

A gap non-union in various conditions has been treated successfully by the Ilizarov method. The gap can be filled up either by an acute shortening and re-lengthening (ASRL) procedure or by an internal bone transport (IBT). We compared the functional and clinical outcome of ASRL and IBT in gap non-unions of the infected tibia.

**Material and Methods:**

A retrospective study was conducted in our department from the data collected in the period between 1997 and 2010. There were 86 cases of infected non-union of the tibia, in patients of the age group 18 to 65 years, with a minimum two-year follow-up. Group A consisted of cases treated by ASRL (n=46), and Group B, of cases by IBT (n=40). The non-union following both open and closed fractures had been treated by plate osteosynthesis, intra-medullary nails and primary Ilizarov fixators. Radical debridement was done and fragments stabilised with ring fixators. The actual bone gap and limb length discrepancy were measured on the operating table after debridement. In ASRL acute docking was done for defects up to 3cm, and subacute docking for bigger gaps. Corticotomy was done once there was no infection and distraction started after a latency of seven days. Dynamisation was followed by the application of a patellar tendon bearing cast for one month after removal of the ring with the clinico-radiological union.

**Results:**

The bone loss was 3 to 8cm (4.77±1.43) in Group A and 3 to 9cm (5.31± 1.28) in Group B after thorough debridement. Bony union, eradication of infection and primary soft- tissue healing was 100%, 85% and 78% in Group A and 95%, 60%, 36% in Group B respectively. Nonunion at docking site, equinus deformity, false aneurysm, interposition of soft-tissue, transient nerve palsies were seen only in cases treated by IBT.

**Conclusion:**

IBT is an established method to manage gap non-union of the tibia. In our study, complications were significantly higher in cases where IBT was employed. We, therefore, recommend ASRL with an established protocol for better results in terms of significantly less lengthening index, eradication of infection, and primary soft tissue healing. ASRL is a useful method to bridge the bone gap by making soft tissue and bone reconstruction easier, eliminating the disadvantages of IBT.

## Introduction

Non-union complicated by infection, unhealthy soft tissues and deformities are difficult to treat^1^. Various strategies have been described to treat such cases, with aggressive debridement, removal of dead bone, filling up of the gap with antibiotic-impregnated cement, bone grafting and internal fixation with plastic reconstruction, and the application of the Masquelet technique. The number of surgeries for each patient is high, leading to increased morbidity and healthcare burden. The soft tissue scars from prior surgeries also limit the surgical options. The goals of treating an infected non-union are infection eradication, promotion of healing of both soft tissue and bone, limb length restoration and function improvement of the limb. Aggressive radical debridement, bone stabilisation and stimulation of bone healing process are the measures taken to achieve our goals. Radical debridement results in gaps between bony fragments. In the Ilizarov technique, stimulation of healing of bone is done by corticotomy that regenerates new bone without the need for bone grafting. Bifocal strategies like internal bone transport (IBT) or acute shortening and re-lengthening (ASRL) can be used to fill such gaps^2-5^. The major problems associated with bone transport are non-union at the docking site and refracture^3^. ASRL is a powerful technique for bridging soft tissue and bone defects. Restoration of limb length and function can be achieved in a single procedure without the need for further reconstructive procedures. Acute shortening results in an inherently stable pattern of the fracture allowing the patient to bear weight soon after surgery^6^.

Studies have shown ASRL to be better than IBT both in terms of a better radiological outcome and a lower rate of complications, with bone grafting of docking site in all these cases to facilitate union^7^. This study was done to compare the clinical and functional outcome of ASRL and IBT in treating gap non-unions without the use of additional bone grafting techniques.

## Materials and Methods

This retrospective study was based on data retrieved and reviewed from hospital records, after obtaining hospital ethical committee approval, of cases with gaps after debridement and non-union of an infected tibial fracture. Ninety-six patients (72 males and 24 females) treated between 1997 and 2010 in our department, operated in a single unit by the same group of surgeons, in the age group 18 to 65 years, with a minimum two-year follow-up, were enrolled into the study. Seventy-two cases occurred following road traffic accident (RTA), sixteen following assault and eight cases following a fall. Sixty-nine cases were had non-union following treatment for an open fracture (Gustillo type III A and B) and twenty-seven cases following infected osteosynthesis of closed fracture [plate (28) / lag screws with plaster (10) /IM nail (46) /external fixator (10) /primary Ilizarov (2)].

Open fractures (n=69) were initially treated with thorough wound debridement and stabilised with an external fixator or internal fixation either primarily or two weeks after primary external fixation. The wound was closed by split skin grafts in 23 cases, and with myo-cutaneous flaps in 6 cases. Forty cases were allowed to heal by secondary intention.

Diagnosis of non-union was made only after a minimum of 6 months of treatment or if there were no signs of healing in radiographs repeated at monthly intervals and the operating surgeon felt that progress to union was absent. The cases either had exposed bone that had been devoid of vascularised periosteal coverage for more than six weeks or had purulent drainage and positive bacteriological culture from the depths of the wound. We excluded cases with hypertrophic nonunion or were lost to follow up before the completion of two years of treatment or who died in the course of treatment because of comorbidities. Ten cases were excluded.

All the cases of infected non-union were classified as per Rosen into three types depending on the status of infection: Type 1, draining with purulent discharge; Type 2, active non-draining but with abscess and fever; Type 3, quiescent without drainage or symptoms for three months or more. The same classification was utilised, with further modification in treatment strategies, to offer the benefits of Ilizarov (Table I).

**Table I:** Showing Classification of Infected non-union and extent of debridement done

	Active Draining	Active Non-Draining	Quiescent
	(Type 1)	(Type 2)	(Type 3)
Draining Sinus	Present	Not Present	Not Present
Sequestration	Extensive	Moderate	Mild to nil
Extent of debridement	Extensive	Moderate	Nil or minimum when implant had to be removed

All active non-union cases (73 cases) were treated with debridement to eradicate infection. Active draining cases (n=53) required extensive debridement, whereas active non-draining cases (n=20) required relatively less extensive debridement. Cases in the quiescent group (n=13) did not require debridement except in a few situations where the implant was to be removed. Type 1 and 2 after debridement and Type 3 cases with or without debridement would finally be labelled as atrophic non-union with a gap, Type B of the Association for the Study and Application of the Method of Ilizarov (ASAMI)^2^ where B1 referred to the length of the limb maintained with a bone gap, B2 to the segments in contact with limb shortening, and B3 to a combined shortening with the defect. The gap was defined as present when there was a loss of length or intercalary tissues exceeding 4cm.

**Fig. 1:**
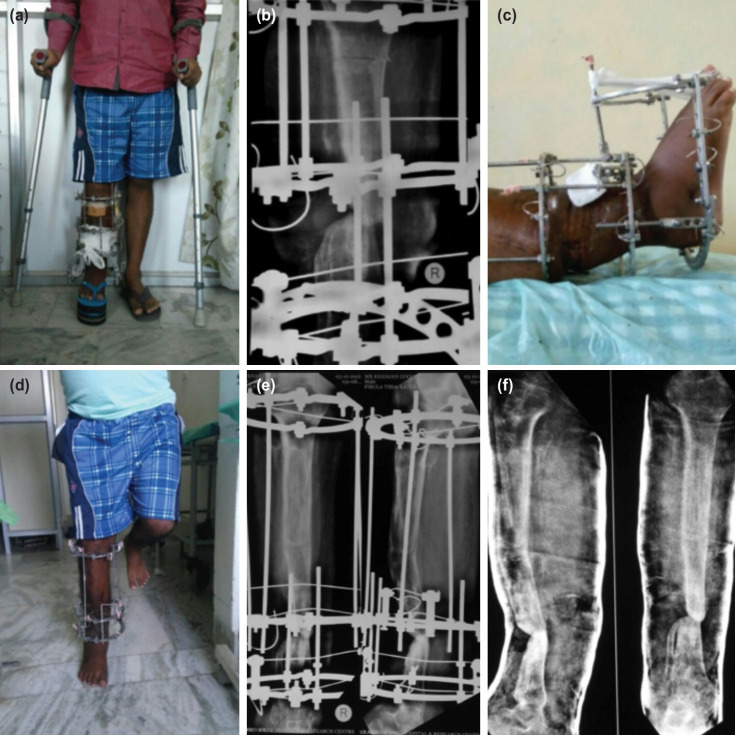
(a) Clinical picture of a patient treated with ASRL. (b) Post-op Radiograph showing docking site and corticotomy being done. (c) Clinical picture showing docking site with foot-frame. (d) Clinical picture during follow-up (e) Radiographs at follow-up showing regenerate and docking site union. (f) Radiographs after Ring removal and Cast immobilisation.

**Fig. 2:**
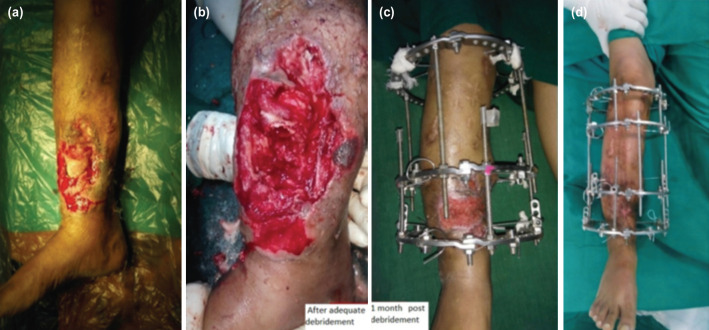
(a) A case of non-union following Type III B open fracture. (b) After debridement with a bone gap of 8cm. (c) One month post-op picture after ASRL showing wound healing by secondary intention. (d) At follow-up for ring removal.

**Fig. 3:**
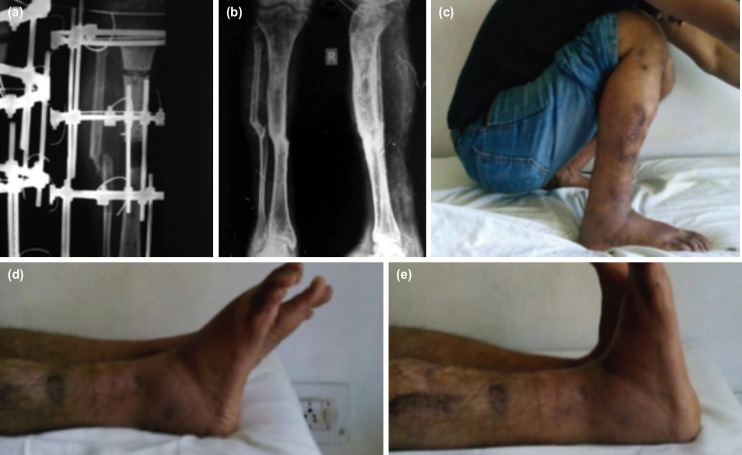
(a) Radiograph during IBT of a case of infected non-union showing regenerate. (b) Final follow-up of the same case after ring removal. (c) Clinical picture at final follow-up showing ROM of knee joint. (d) Clinical picture at final follow-up showing ankle plantar flexion. (e) Clinical picture at final follow-up showing ankle dorsiflexion.

**Fig. 4:**
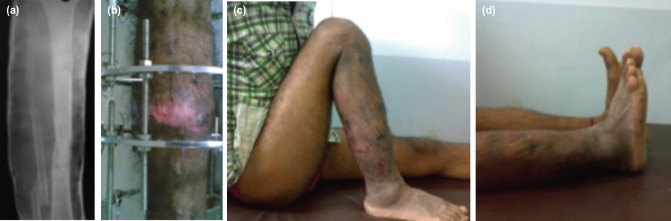
(a) Final follow-up radiograph of a case treated by IBT. (b) clinical picture before ring removal. (c) Clinical picture showing ROM of knee flexion. (d) clinical picture showing ROM of ankle plantar flexion. (e) Clinical picture showing ROM of ankle dorsiflexion.

**Fig. 5:**
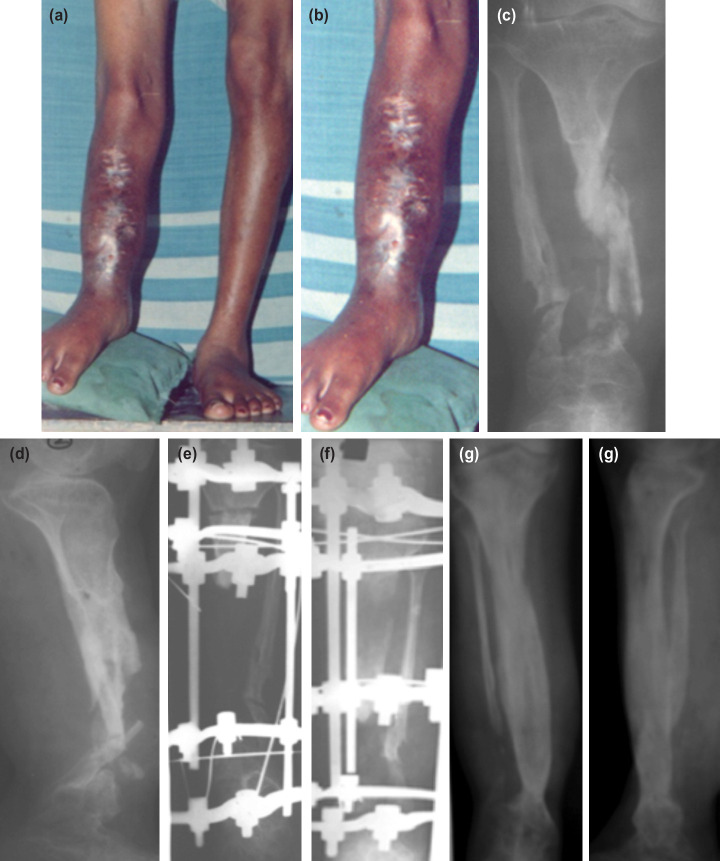
(a) Pre-op clinical photo of a case of infected non-union tibia with LLD. (b) Magnified image showing infected part with sinuses. (c) Pre-op AP view radiograph showing infected non-union. (d) Pre-op lateral view radiograph showing infected non-union. (e) After radical debridement and corticotomy with massive bone gap. (f) Subsequent radiograph during bone transport showing regenerate bone. (g) Final follow-up AP view radiograph showing union and regenerate. (h) Final follow-up Lat view radiographs showing union and regenerate.

The limbs were stabilised with either all wire conventional or hybrid Ilizarov ring fixators. Four ring assembly was used with two rings on either side of the gap. Additional foot frames were used in cases of distal metaphyseal fractures. ASRL strategy was followed during the entire nine years duration of the study period (Fig. 1 and 2), and IBT strategy was followed during the first six years of the study period (Fig. 3, 4 and 5).

**Fig. 6:**
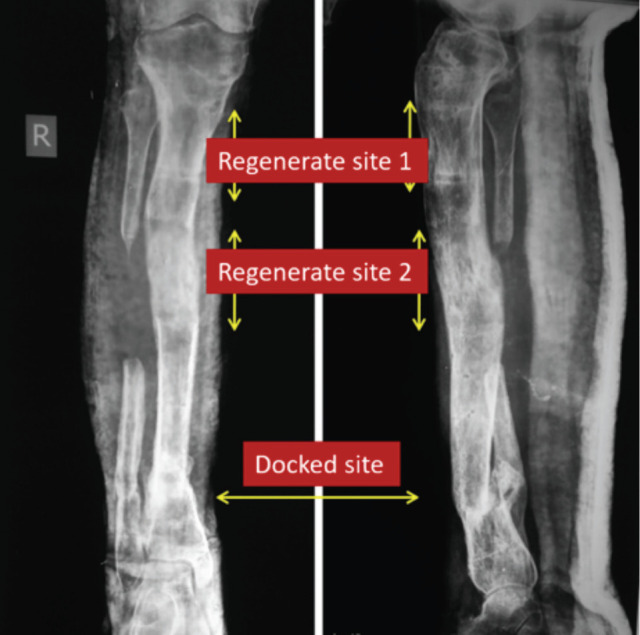
Trifocal osteosynthesis with 4cm regenerate at both side of docking.

**Fig. 7:**
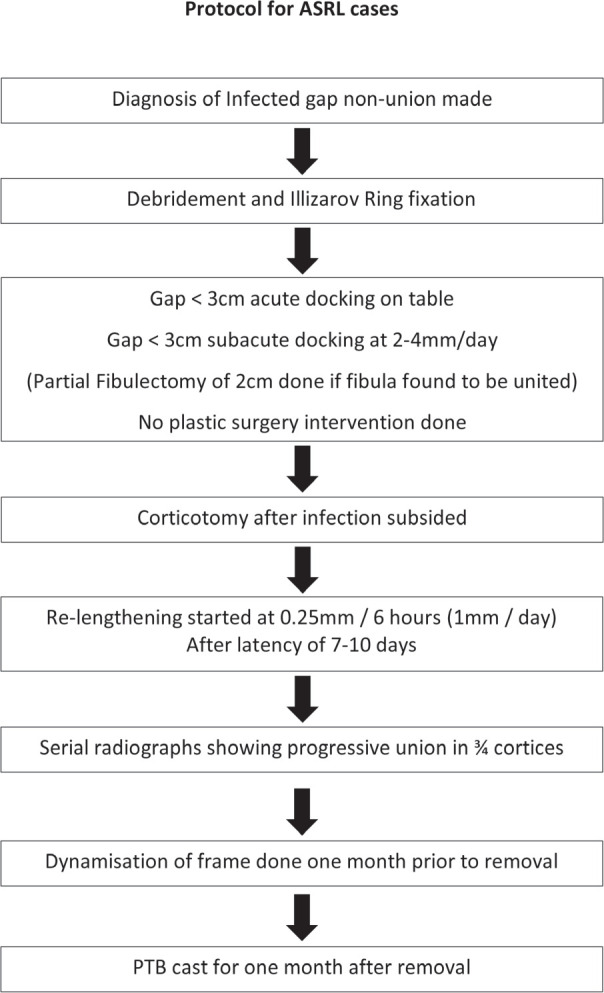
Protocol for Acute Shortening and Re-lengthening.

**Fig. 8:**
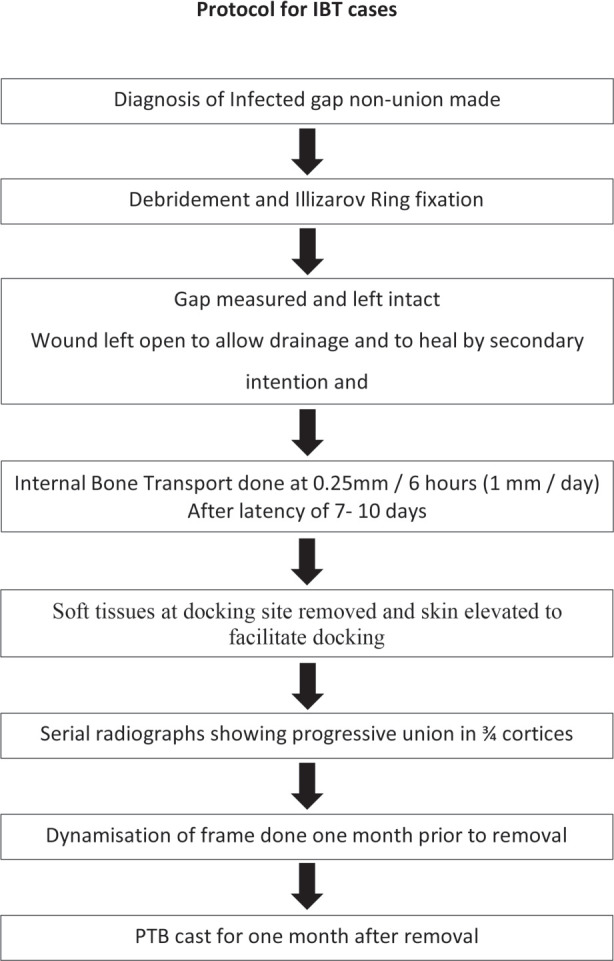
Protocol for Internal Bone Transport.

Corticotomy was done only after infection subsided in all cases. Healthy granulation tissue locally at the wound site and normal haematological parameters were pre-requisites. It was usually done at three to four weeks after stabilisation in the majority of cases. Proximal corticotomy was done in most of the cases except in five cases (three of IBT and two of ASRL) and trifocal osteosynthesis and distal corticotomy with distal to proximal bone transport in four cases (Fig. 6). We followed our Institutional protocol for ASRL (Fig. 7) and IBT (Fig. 8) in all cases.

After docking, in both groups, the site was compressed 1/4th every 3rd day until four weeks. Weight-bearing was started early, as the pain subsided and mobilisation of adjacent joints was encouraged. Shoe rise was given to facilitate easy walking, the height of which was subsequently reduced fractionally at each visit to facilitate weight-bearing. Physiotherapy to improve the muscle strength and range of motion of ankle and knee was advised. Patients were discharged once the wound healed. They or their attendants were taught to take care of pin tracts by washing with normal saline, were encouraged to take a bath on a ring, and perform distraction or compression. They were asked to come back early if there was pain or discharge.

On every follow-up, usually on every 4th week, the tension of wires was checked and adjusted accordingly. Pin sites were checked to rule out any pin-tract infections of significance. Nuts were checked for any loosening. Clinical and radiological assessments were done by one of the operating surgeons and were confirmed by the senior-most surgeon in all cases.

Removal of the apparatus was considered when union in three out of four cortices was seen in a radiograph. Dynamisation was done by the staged loosening of the nuts in threaded rods both at the regenerate and docking sites, every week, for four weeks before the final removal of the apparatus. After removal of the apparatus, the limb was further supported with PTB cast for one month. Final follow up was done for each case at 12 months after the removal of the apparatus.

Statistical analyses were performed using SPSS version 20. Results were expressed as mean ± standard deviation for continuous variables and as number (%) for categorical data. Comparisons between two quantitative variables between two groups were made, using student’s t-test. Fisher exact test and chi-square test were applied where they were applicable. A p-value of less than 0.05 was considered significant.

## Results:

Eighty-six cases of infected gap non-union of the tibia were included in our study as they had completed a minimum of two years of follow-up. Ten cases were excluded. The gap non-union of the tibia was either the consequence of bone loss due to debridement of infected osteosynthesis of open cases (n=60) or closed fractures (n=26). The initial bone loss was 3cm to 8cm in Group A (n=25) and 3cm to 9cm in Group B (n=25) (Table II). Most of the cases (approx. 60 %.) were in Rosen classification type I followed by type II^8^.

**Table II:** Pre-op patient demographics

	Group A (ASRL)	Group B (IBT)
Total no of included cases	46	40
	(M: F= 33:13)	(M: F= 31:9)
	Mean age=36.92 ±12.16	Mean age =37.83±12.35
Grade of infection (Rosen classification)	Type 1 =28 (60.86%)	Type 1 =25 (62.5%)
	Type 2=12 (26.08%)	Type 2=8 (20.00%)
	Type 3= 6 (13.04%)	Type 3= 7(17.50%)
Bone gap (cm)	3-8	3-9
	Avg (4.77 ±1.43)	Avg (5.31±1.28)

Twenty out of 86 patients were suffering from co-morbidities like diabetes mellitus (n=6), anaemia (n=15) and hypoalbuminemia (n=5). The average number of surgeries performed before intervention for non-union were five; initial debridement, ex-fix application, re-debridement, plastic intervention and internal fixation for open fractures. It was three for closed fractures: internal fixation, debridement and plastic intervention. There were soft tissue contractures and scarring with equinus in 10 cases before treatment, but no neurovascular injury was noted. Intra-operative cultures of debrided samples showed that the most common organism was Staphylococcus aureus followed by klebsiella species, and appropriate systemic antibiotics were started till the infection was under control.

Parameters analysed were bone healing, functional results, and complications, according to Paley and ASAMI^9-11^. Lengthening index (healing index or regeneration index) was calculated by dividing the frame-keeping period (days) by the length of the regenerated bone (cm)^12, 13^. It was used to compare with the indices in other studies.

**Figure:**
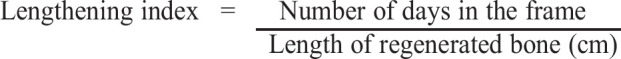


The functional result was evaluated according to ASAMI criteria based on the five parameters; of significant limp, equinus deformity of the ankle, soft tissue dystrophy, pain and inactivity^9^. Complications that occurred during or after surgery and distraction-compression, were evaluated according to the Paley working classification^9^.

Bony union, eradication of infection and primary soft tissue healing was 100%, 85% and 78% in Group A and 92%, 60%, 36% in Group B respectively. The result was highly significant in terms of primary soft-tissue healing, and significant in eradication of infection (Table III) Complications like non-union at docking site, equinus deformity, false aneurysm, interposition of soft tissue, and transient nerve palsy were seen only in group B (Table IV).

**Table III:** Comparison of results between ASRL and IBT

Parameter	Group A ASRL (n=46)	Group B IBT (n=40)	Significance
Bony union	100%	92%	p = 0.0587, NS
Eradication of Infection	85%	60%	p = 0.0043, S
Primary soft tissue healing	78 %	36%	p < 0.001, HS

**Table IV:** Evaluation of outcome in both groups

	GROUP A ASRL (n=46)	GROUP B IBT (n=40)	Significance
**BONE HEALING**			
Excellent	10 (21.73%)	6 (15%)	p = 0.557, NS
Good	24 (52.17%)	18 (45%)	
Fair	8 (17.39%)	10 (25%)	
Poor	4 (8.69%)	6 (15%)	
Lengthening index	38.37 ± 2.35	43.54 ± 3.37	p < 0.001, HS
**FUNCTIONAL RESULT**			
Excellent	8 (17.39%)	5 (12.5%)	p = 0.059, NS
Good	26 (56.52%)	18 (45%)	
Fair	10 (21.73%)	12(30%)	
Poor	2 (4.34%)	5 (12.5%)	
**COMPLICATIONS**			
Nonunion at docking site	0 (0%)	2 (5%)	p = 0.1249, NS
Persistence of infection	7 (15.21%)	16 (40%)	p = 0.0096, S
Equinus deformity	0 (0%)	3 (7.5%)	p = 0.058, NS
False aneurysm	0 (0%)	1 (2.5%)	p = 0.280, NS
Soft-tissue interposition	0 (0%)	10 (25%)	p = 0.0003, S
Delayed consolidation	2 (8%)	8 (20%)	p = 0.023, S
Transient nerve palsy	0 (0%)	2 (5 %)	p = 0.1249, NS

There was no significant difference between the groups in terms of bone healing, functional outcome and complications of non-union at docking site, equinus deformity of the ankle, false aneurysm formation and transient nerve palsies. The only significant difference in complications between the groups was seen in terms of persistence of infection, softtissue interposition and delayed consolidation.

A false aneurysm was seen in one (2.5%) case treated with IBT with a trifocal osteosynthesis and distal to proximal bone transport. Towards the end of transport when docking was about to be achieved, there was profuse bleeding. An immediate exploration was done, where the bleeding from the lateral peroneal artery was identified close to the proximal third of tibia and ligated, and the wire replaced.

Equinus deformity occurred in three cases (7.5%) of bone transport with gaps more than 5cm. Transient nerve palsy of the deep peroneal nerve occurred in two cases (5%) treated by IBT, with new onset paraesthesia over the medial foot and foot drop. One case was being treated by trifocal osteosynthesis. and the bone transport was stopped immediately and reversed by 1mm/day. Eventually, the palsy recovered, and IBT was started again but at a slower rate of ½ mm a day. Stretching of the nerve leading to neuropraxia could be the cause of such transient palsy.

Delayed consolidation of regenerate occurred in two (8%) cases treated with ASRL and eight (20%) cases treated by IBT. Persistence of infection was found to be associated with IBT, and the regenerate quality improved once infection subsided with prolonged systemic antibiotics and repeated debridement to remove the infectious load. Temporary stoppage of distraction was done during active infection to avoid the loss of regeneration. Once the infection was adequately controlled as the bone gap was more than 3cm in three cases, repeated corticotomy was done at other end of the bone. The average duration of treatment of the cases was dependent upon the length of the gap. In the ASRL mode of treatment, the average lengthening index was 36 to 40 days, and for IBT, it was 40 to 46 days.

The average number of surgeries performed in both group were four for ASRL and five for IBT. They were debridement, Ilizarov fixation, re-debridement if needed, with acute or subacute docking for ASRL cases; and fibulectomy, corticotomy, docking with or without readjustment and removal of interposed soft tissues for IBT cases. Repeated debridements were necessary in more cases of IBT than ASRL to control infection.

## Discussion

Infected non-union makes the life of the patient miserable^14^. Long-standing infected non-union has problems of extensive scarring, unpredictable osteomyelitis, adjacent joint stiffness, deformity, bone gap, and limb length discrepancies, among others^15^. The aims of treating infected non-union are to eradicate the infection, promote healing of both soft tissue and bone, restore limb length and restore limb function. These require aggressive radical debridement, bone stabilisation and stimulation of the bone healing process. In the Ilizarov technique, stimulation of the healing of bone is done by corticotomy that regenerates new bone without the necessity of bone grafting.

Debridement, an essential initial step in managing infected non-union of long bones, leads to a gap. The two bifocal Ilizarov strategies, either IBT or ASRL, used to fill up the gap, have their advantages and disadvantages. Internal bone transport has advantages of simultaneous correction of the bone gap and limb shortening. Also, as new bone formation occurs in the gap, the actual distraction required is less. It also helps in the improvement of local soft tissue conditions, thereby creating ease in physiotherapy. However, there are serious disadvantages, especially resulting in docking site problems including non-union and refracture^3^, limb axis deviation, translation of bone fragments, soft tissue interposition, joint contracture and joint subluxation^16^. In long-standing non-union, the bone ends are usually osteopenic. The osteopenia progresses during the distraction period. Therefore, at the end of distraction, the constant tension stress is absent, causing unstable fixation at the docking site^4^. The Ilizarov apparatus stays for a period longer than required for comparable lengthening in patients, as healing of the target site begins only after intercalary lengthening of the fragment is complete^17^.

Acute limb shortening and re-lengthening is another technique for bridging soft tissue and bone defects and restoration of limb length and function in a single procedure without the need for further reconstructive procedures in most cases. Although based on the Ilizarov techniques, it eliminates the problems frequently encountered with internal bone transport by converting a complicated limb reconstruction into a relatively simpler procedure. Acute shortening results in an inherently stable pattern of the fracture. Having immediate stability allows the patient to walk and bear weight soon after surgery. In acute cases, this active and functional management can shorten the time of treatment and reduce costs and absence from work^16^.

Acute limb shortening and re-lengthening had been described for the management of longitudinal bone defects with or without soft-tissue loss due to different etiologies^5, 18-22^. A study by Tetsworth *et al*^7^ comparing the results of bone transport and ASRL in managing infected non-union with bone defect demonstrated excellent results with both the techniques, with ASRL having a better radiological outcome and a lower rate of complications than IBT. They routinely performed bone grafting of the docking site to enhance union, whereas, in our study, we treated all cases without additional bone grafts.

ASRL strategy had better control of infection than IBT. We hypothesise that ASRL can be considered as the advancement of a large vascularised osteo-myo-cutaneous flap to close the defect, replacing all devitalised and infected tissues with local healthy tissue without the need for additional plastic intervention. With gap closure, the potential dead space is eliminated, reducing the potential for infection in the environment. With the bone and soft-tissues in contact, the healing process starts from Day 1 of surgery with better soft tissue scores than IBT cases.

Saleh and Rees compared the results of the treatment of bone defects by bone transport with those of acute limb shortening followed by lengthening. They obtained excellent results in 12 patients (75%) and good results in four (25%). They found a shorter treatment time and fewer complications with limb shortening and re-lengthening. They recommended that acute shortening should be considered for tibial defects of ≤ 3cm and femoral defects of ≤ 5cm^5^. Sen *et al.* described the results of acute shortening and re-lengthening in the acute treatment of grade III open tibial fractures with osteo-cutaneous loss in 24 patients using the evaluation system of Paley *et al*^9^. The bone results in their series were excellent in 21 and good in three. The functional result was excellent in 19, good in four and fair in one patient. No bone or skin grafts was used^21^.

Flap closure results in decreased drainage, which is against the principle of keeping the wound open to allow adequate drainage of infected material. There can be apprehension regarding the persistence of infection in ASRL cases, but our results showed that ASRL cases had better infection control than IBT. The false aneurysm, which was a complication in a case of distal to proximal bone transport following distal corticotomy, could be attributed to an impingement of a branching point of the lateral peroneal artery from below during transport. Similarly, equinus deformity was commonly seen in cases of IBT. We hypothesised that the stretching of the gastrosoleus could lead to progressive equinus deformity. Foot frames should be added during the distraction phase to prevent equinus. Post-operatively active movement of the ankle should be encouraged with rubber sandal fixed to the frame with elastic or rubber bands. In our series, we chose ASRL during the later period of our study in the last nine years in an attempt to avoid the complications encountered earlier with IBT.

## Conclusion

IBT is an established method to manage gaps created in infected non-union of the tibia after debridement and bone loss. We have found in our study that the persistence of infection, soft tissue interposition and delayed consolidation, were significantly high in cases where IBT was employed. We, therefore, recommend that ASRL with an established protocol can be employed for a significantly less lengthening index, eradication of infection, and primary soft tissue healing. ASRL is a useful method to bridge the bone gap, as the soft tissue and bone reconstruction is easier by eliminating the disadvantages of internal transport. The formation of new bone in the gap in IBT makes the actual distraction needed to be much less, and is an advantage for IBT, but is seen in only a few cases, and not seen uniformly in all cases.

Our study is a retrospective non randomised one based on hospital records, with IBT in the earlier phase and ASRL in the later phase. A prospective randomised study of both techniques would allow for a better evaluation of the outcome.
